# Hypomagnesaemia associated with long-term use of proton pump inhibitors

**DOI:** 10.1093/gastro/gou054

**Published:** 2014-08-19

**Authors:** James Wei Tatt Toh, Evonne Ong, Robert Wilson

**Affiliations:** ^1^General Surgery, Liverpool Hospital, University of New South Wales, Liverpool, New South Wales, Australia,; ^2^University of New South Wales Clinical School, Liverpool, New South Wales, Australia and; ^3^Upper Gastrointestinal Surgery, Liverpool Hospital, University of New South Wales, Liverpool, New South Wales, Australia

**Keywords:** Proton pump inhibitors, hypomagnesaemia, hypocalcaemia

## Abstract

Hypomagnesaemia and associated hypocalcaemia and hypoparathyroidism have been increasingly recognised as rare long-term side-effects of proton pump inhibitors (PPIs). The PPIs may inhibit active magnesium (Mg) absorption by interfering with transcellular transient receptor potential melastatin-6 and -7 (TRPM 6 and 7) channels. More recent cell culture studies have suggested concomitant inhibition of passive Mg absorption by omeprazole. After being treated with a range of PPIs, the four patients in our case series developed hypomagnesaemia, which responded to withdrawal of therapy and initiation of Mg replacement. Their clinical course and management demonstrate key aspects of hypomagnesaemia associated with long-term use of PPIs.

## INTRODUCTION

Since 2006 there have been approximately 40 reported cases of proton-pump inhibitor (PPI)-induced hypomagnesaemia (PPIH) [1]. In March 2011, the US Food and Drug Administration (FDA) issued a safety announcement, including hypomagnesaemia as a long-term side-effect of PPI based on accumulating evidence [2–5]. The Australian Therapeutic Goods Administration (TGA) released a similar alert in June 2011.

Hypomagnesaemia with PPI use is uncommon, occurring in less than 1% of all PPI-induced side-effects voluntarily reported to the FDA [6]. The mechanism by which this occurs is still being investigated. PPIs may decrease intestinal magnesium (Mg) absorption by interfering with both active [transient receptor potential melastatin (TRPM) protein channels] and passive (paracellular pores) absorption [6, 7]. PPIs bind irreversibly to the parietal cell H/K adenosine triphosphate (ATP) pump and inhibit gastric acid production by 95%. Congenital defects, such as mutations in TRPM channels, may be responsible for hypomagnesaemia in some patients [8]. Another potential explanation is PPI revealing otherwise subclinical hypomagnesaemia. The true incidence of hypomagnesaemia in patients on long-term PPIs is unclear [9]. Hess *et al.* indicated that PPIH occurred after a median 5.5 years of PPI use, with onset ranging from 14 days to 13 years after commencement of use [10]. Discontinuation of PPIs resulted in rapid recovery of serum magnesium within 4 days. Magnesium recovery did not occur with oral magnesium replacement alone, nor with repeated intravenous (i.v.) magnesium infusions. Hypomagnesaemia rapidly recurred on resumption of PPI therapy with PPI, but not if H2 antagonists were used as alternative acid suppression [10]. This observed challenge, discontinuation and re-challenge phenomenon with PPIs indicates that PPIH is a real syndrome [1–4, 6, 8–11].

Hypomagnesaemia associated with PPIs can cause a range of symptoms of varying incidence, including tremor of the extremities, convulsions (40%) [12], muscle cramps and spasms (20%), weakness and lethargy (30%) [13], tetany (17%) [14], loss of consciousness [15], numbness, anxiety, hallucinations, agitation (20%), dizziness and nausea (36%), carpopedal spasm associated with hypoparathyroidism and hypocalcaemia, signs such as Trousseau and Chvostek sign [16], QT prolongation, ataxia, concomitant hypokalaemia with electrocardiogram (ECG) changes and arrhythmias (30%) [10, 17]. Tetany or neuromuscular irritability can be related to co-existent hypomagnesaemia and hypocalcaemia.

## CASE SERIES

### Case 1

An 84-year-old male presented with dysphagia for six months. He was investigated and diagnosed with poorly differentiated oesophageal squamous cell carcinoma. Positron emission tomography (PET) scan showed no metastases. He had a history of diet-controlled diabetes, hypertension, prostate cancer and was an ex-smoker. His medications included trandolapril, esomeprazole and atorvastatin. He underwent definitive chemoradiotherapy, receiving 50.4 Gy over 28 fractions and carboplatin and paclitaxel from 11 March 2013 for a period of one month. There was no evidence of chemotherapy-induced Mg wasting nephropathy.

He had been prescribed esomeprazole for gastro-oesophageal reflux disease (GORD) for seven years. His Mg level was 0.33 mmol/L (normal range: 0.7–1.0 mmol/L) on 4 June 2013. Apart from muscle cramps in his lower limbs, he had no other symptoms of hypomagnesaemia. He had concomitant hypocalcaemia (corrected Ca 2.01 mmol/L; normal range 2.10–2.60 mmol/L) but his parathyroid hormone (PTH) level was paradoxically normal (3.9 pmol/L; normal range 1.5–7.1 pmol/L). His vitamin D level was 69 nmol/L. Both his urinary Mg and Ca excretion were low (0.7 and 0.1 mmol/24 hours, respectively), indicating preserved renal re-absorption of tubular Mg and Ca. Normal range renal Mg excretion is 2.5–6.5 mmol/24 hrs, and normal range renal Ca excretion is 2.5–7.5 mmol/24 hrs.

**Figure 1:**
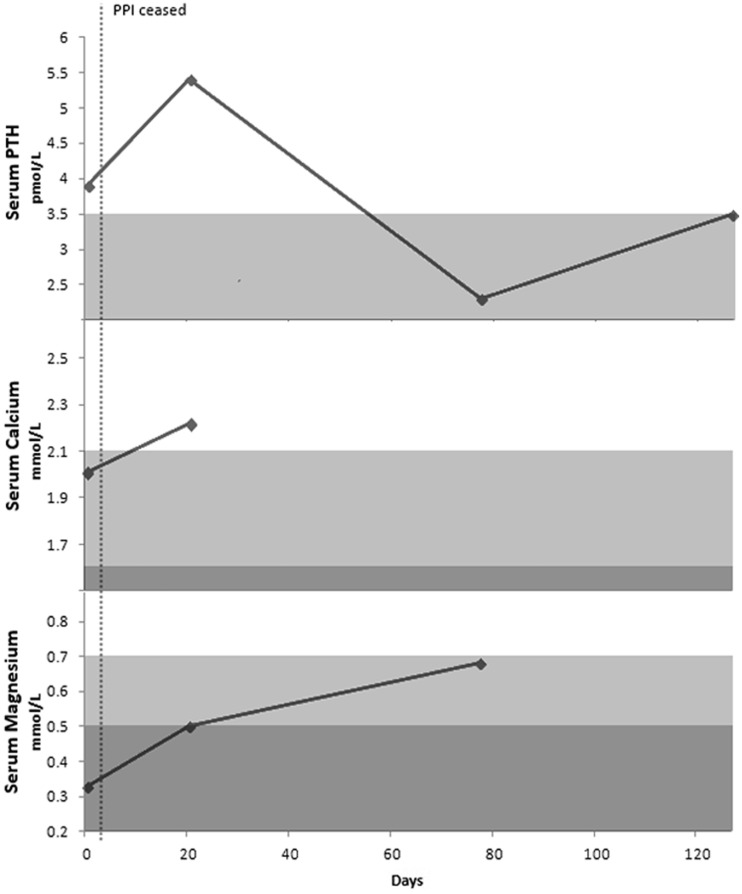
Serum parathyroid hormone (PTH), calcium and magnesium for Case 1.

He was started on Mg replacement (1 g magnesium/day) and esomeprazole was stopped on 7 June 2013. He was started on ranitidine. His Mg levels improved to 0.5 mmol/L within 20 days of stopping esomeprazole and his calcium (Ca) levels improved to 2.22 mmol/L. However, after stopping esomeprazole, the patient developed minor acid reflux and eructation on ranitidine. Due to his hypomagnesaemia, it was decided not to restart a PPI. After 3 months, his serum levels of Mg continued to improve to 0.68 mmol/L and his vitamin D level remained stable at 65 nmol/L. His Ca and Mg urinary excretion had improved to 2.2 and 0.5 mmol/24 hrs, respectively (Figure 1).

### Case 2

An 83-year-old female was diagnosed with metastatic jejunal gastrointestinal stromal tumour (GIST) confirmed on laparoscopic peritoneal biopsy on 5 June 2012. She was treated successfully with imatinib 400 mg daily *per os*. She had Type 2 diabetes, hypothyroidism and hypertension. Her medications included irbesartan, metformin, iron, acarbose, vitamin D, gliclazide, lisinopril, prochloperazine, amlodipine, atorvastatin and thyroxine.

On 25 May 2012, she complained of muscle cramps, constipation and lethargy. She had been taking pantoprazole for five years for epigastric pain. Her Mg and Ca levels were low (Mg 0.54 mmol/L; Ca 2.09 mmol/L). PTH was inappropriately low (3.6 pmol/L) for her serum calcium level. At the time, her vitamin D level was 124 nmol/L.

**Figure 2:**
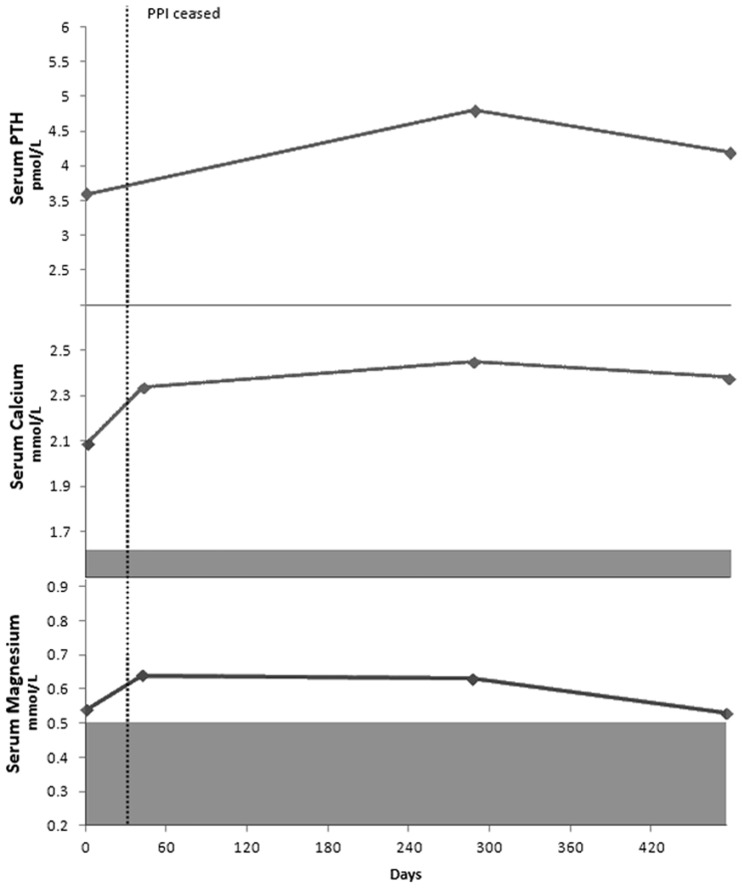
Serum parathyroid hormone (PTH), calcium and magnesium for Case 2.

Her pantoprazole was stopped and she commenced on 1.5 g magnesium per day. After stopping pantoprazole, her leg cramps, constipation and fatigue improved and her Mg level rapidly improved within days to 0.64 mmol/L. At this time, her Ca level improved to 2.34 mmol/L. After two months, her Mg replacement was reduced to 1 g per day. After stopping the pantoprazole and receiving Mg replacement, her Mg and Ca urinary excretion increased from 2.1 and 1.9 mmol/24 hours to 5.9 and 5.4 mmol/24 hrs, respectively (Figure 2). Her vitamin D level remained within normal range at 72 nmol/L.

### Case 3

A 72-year-old male underwent a colonoscopy and polypectomy on 7 December 2011, including polypectomy on a large sessile caecal adenoma. The procedure was complicated by post-operative pain and a computed tomography (CT) scan showed inflammation in the right iliac fossa, but no perforation. He was managed non-operatively with intravenous antibiotics. He then developed diarrhoea (stool cultures negative for pathogens including *Clostridium difficile* toxin) which responded to probiotic treatment. He had Type 2 diabetes and other significant comorbidities including osteoarthritis, peptic ulcer, ischaemic heart disease, asthma, atrial fibrillation and obesity. Medications included warfarin (for atrial fibrillation and myocardial infarct), clopidogrel, metoprolol, atorvastatin, gliclazide, symbicort, bricanyl, metformin, ranitidine, allopurinol and temazepam.

Two months after being discharged, he re-presented to the emergency department with severe generalized weakness, inability to stand from sitting and shortness of breath. His serum Mg and Ca levels were found to be extremely low (0.27 and 1.80 mmol/L, respectively) on 20 February 2011, with a vitamin D level of 72 nmol/L. His PTH was 12.4 pmol/L: this may have been elevated due to PTH being an acute phase reactant in acute inflammation, as subsequent PTH levels were low–normal with persistent hypomagnesaemia. At the time, he had been on pantoprazole for at least six years. He was given intravenous Mg 40 mmol and intravenous Ca and admitted to the high-dependency unit. His muscle weakness, malaise and lethargy responded rapidly to intravenous Mg and Ca replacement. He was later discharged on 2 g magnesium per day, caltrate 600 mg twice daily and ostelin 25 μg twice daily. His pantoprazole was not ceased, as PPIH was not recognized during this admission. His serum magnesium levels remained low, and his muscular weakness and shortness of breath recurred, despite high dose oral Mg replacement.

**Figure 3:**
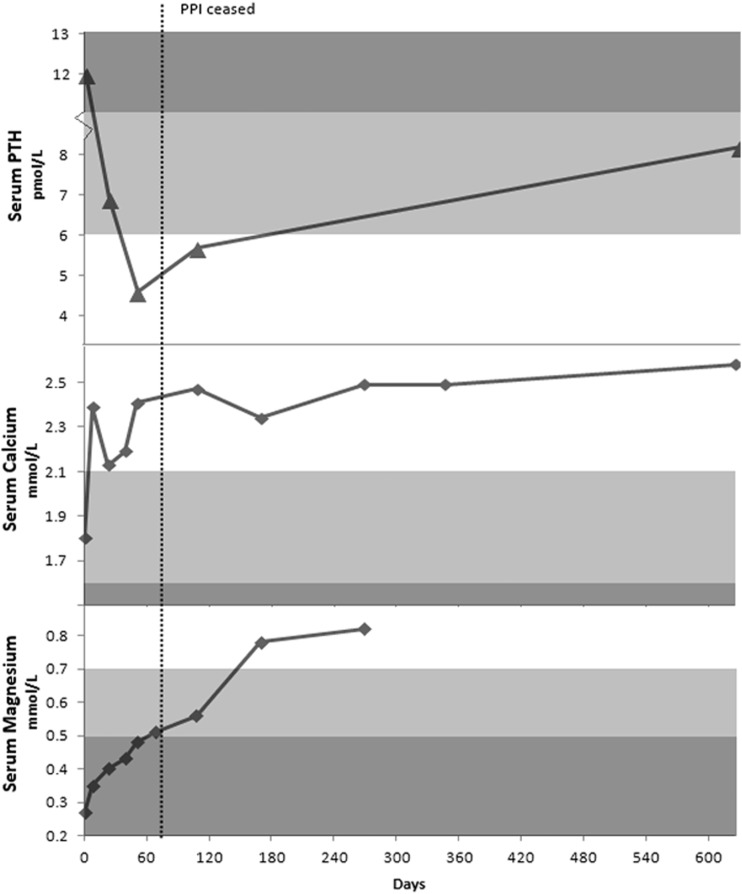
Serum parathyroid hormone (PTH), calcium and magnesium for Case 3.

On 30 April 2011, he was changed from pantoprazole to ranitidine. Two weeks later, his Mg dose was increased to 6 magnesium tablets per day. He was also started on oral sunflower seeds. His dose of ranitidine was increased to 300 mg daily on 9 November 2011, due to worsening reflux and indigestion. After his pantoprazole was ceased and Mg replacement commenced, his urinary excretion of Mg and Ca improved (from 2.2 mmol and 1.4 mmol/24 hours to 4.2 mmol and 2.4 mmol/24 hours respectively). His serum calcium (2.47 mmol/L), Mg (0.56 mmol/L) and PTH (5.7 pmol/L) also normalized (Figure 3). Vitamin D levels remained stable (102 nmol/L) throughout treatment.

### Case 4

This case was a 68-year-old female with post-prandial GORD attributed to a hiatus hernia. She was diabetic and also had a history of steatohepatitis, hypertension, hypothyroidism, post-traumatic stress disorder, benign positional vertigo, osteoarthritis and aortic valve calcification. Medications included aspirin, risedronate sodium, esomeprazole, irbesartan/hydrochlorothiazide, betamethasone, metformin, atorvastatin, glyceryl trinitrate spray, thyroxine, oestriol cream and lercanidipine.

On 19 February 2011, she presented with right-upper quadrant pain and was diagnosed with cholelithiasis. She had been taking esomeprazole for five years for GORD. Her Mg level was incidentally found to be low (0.43 mmol/L). The corrected Ca was normal at 2.37 mmol/L. Urinary Ca and Mg excretion were low at 0.2 and 0.7 mmol/24 hours, respectively. She reported leg cramping and lethargy.

**Figure 4:**
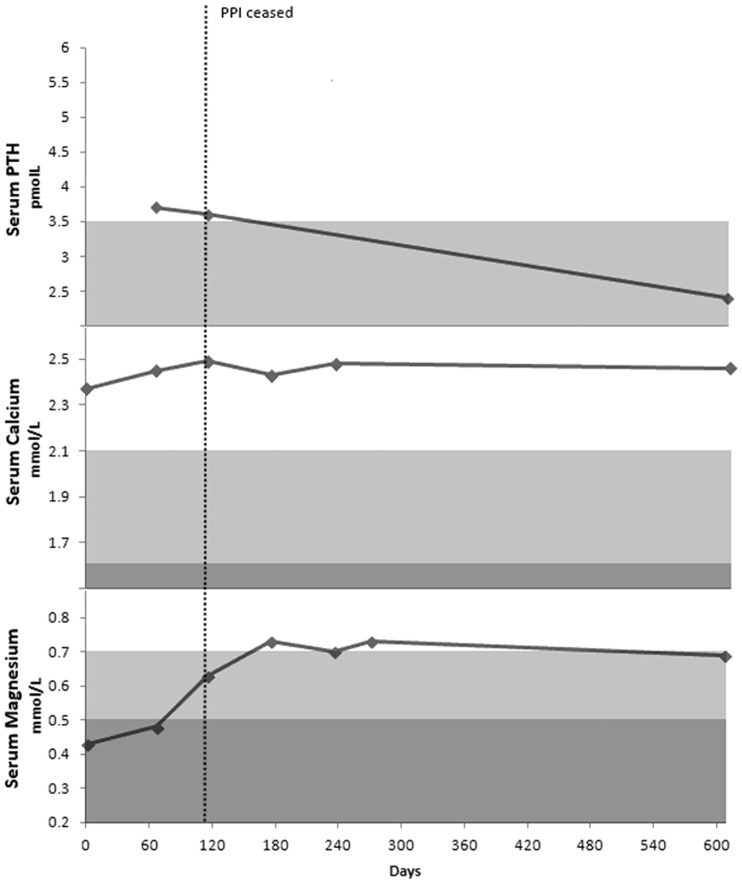
Serum parathyroid hormone (PTH), calcium and magnesium for Case 4.

Her esomeprazole was changed to ranitidine and she was started on Mg supplements on 21 October 2011. Her serum Mg and Ca (0.73 and 2.43 mmol/L) and urinary Mg and Ca excretion improved to 2.2 mmol and 2.2 mmol/24 hour, respectively. At this time, her vitamin D level was 61 nmol/L (Figure 4). Her leg cramps and lethargy resolved, however, she had recurrence of reflux and indigestion with ranitidine and was commenced on mylanta with little improvement. Further investigations, including oesophageal pH manometry and gastroscopy, showed Barrett’s oesophagus with acid reflux and hiatus hernia. She underwent laparoscopic Nissen fundoplication and her acid suppression medication was ceased with resolution of symptoms.

## DISCUSSION

The four patients with PPIH described in this case series add to the accumulating evidence that long-term use of PPIs can cause electrolyte derangement, including hypomagnesaemia and hypocalcaemia. While a range of symptoms has been described in the literature, the most common symptoms observed in our case series included muscle weakness, cramping and lethargy. Two patients who developed hypomagnesaemia were taking pantoprazole (lowest potency) [18], and two patients esomeprazole, which suggests that PPI has a class effect in the aetiology of hypomagnesaemia [1, 18]. This is supported by the observation that substitution of one PPI for another results in recurrence of symptoms of PPIH and electrolyte abnormalities [11, 18]. Patients were referred to a consultant endocrinologist with specific expertise in the management of hypomagnesaemic hypocalcaemia and PPIH, to confirm the diagnosis, exclude other causes and optimize management.

Whilst the case series by Mackay *et al.* showed a female preponderance for PPIH (male = 1, female = 9) [3], our case series did not suggest gender differences. In fact, the FDA data showed males to be at higher risk of PPIH than females, and patients older than 65 years of age were more likely to develop PPIH than their younger counterpart [10]. Only one of the four patients in our case series who developed PPIH were on a diuretic (hydrochlorothiazide), unlike the studies by Mackay *et al.* and Danziger *et al.* [3, 19], which found that PPIH required concomitant diuretic treatment. The median age of diagnosis of PPIH in our case series was 77 years and duration of PPI was 5.75 years, similar to the findings by Hess *et al.* [10].

In our series, PTH was found to be normal in three cases and elevated in one. Three of our cases had associated hypocalcaemia. Hypoparathyroidism associated with PPIH was first reported by Epstein *et al.* [20], but not observed in any of the patients in our study. While none of the patients in our series had hypoparathyroidism, two patients had inappropriately low PTH levels in relation to their hypocalcaemia, suggesting possible functional hypoparathyroidism. Hypomagnesaemia is known to induce hypocalcaemia, due to interference with Ca sensing receptor transduction, inhibition of PTH release, end-organ resistance to PTH, and increased breakdown of PTH into inactive metabolites [20].

Improvements in serum Ca and Mg and resolution of renal conservation of Ca and Mg were demonstrated in all our cases when PPI was stopped and hypomagnesaemia was corrected. In one patient who restarted PPI with oral Mg replacement after one year of persistent reflux on ranitidine, the Mg level did not dramatically fall. In their systematic review, Hess *et al.* found that hypomagnesaemia rapidly recurred on restarting PPIs [10]. Within four days of restarting PPIs, serum Mg levels fell below 0.6 mmol/L in 70% of patients who had previously been treated for PPIH [10]. This reflected the extreme susceptibility to PPIs in selected patients and also their severely depleted body Mg stores [5].

Hypomagnesaemia has also been associated with diabetes and renal Mg wasting [21, 22]. Three of our patients were non-insulin-dependent diabetics on metformin, and one patient had diet-controlled diabetes. However, none had evidence of increased urinary Mg losses on 24-hour urine studies. In fact, all our patients had profoundly suppressed urinary Mg and Ca excretion, which improved after PPI withdrawal and Mg supplementation. This was also seen in other series of PPIH, suggesting that PPIs do not interfere with renal handling of Mg [3, 5, 10, 13, 17, 21–23].

### Magnesium and calcium physiology

Calcium and magnesium homeostasis depends on the balance between intestinal absorption, bone exchange and renal excretion [24]. Intestinal Mg absorption is achieved by active saturable transcellular transient receptor potential melastatin 6 and -7 (TRPM6 and -7) channels and by non-saturable paracellular passive diffusion. TRPM6 and -7 protein channels are responsible for active transport of divalent cations including Mg, Ca, zinc, cobalt, manganese and nickel. The highest affinity is for Mg cations [25]. High cytosolic Mg inhibits the activity of the TRPM6 and -7 cation channels, which provides a negative feedback effect on active Mg absorption in times of Mg surfeit [25].

Calcium is also absorbed by active transcellular transport via transient receptor potential vanilloid 5 and -6 (TRPV5 and -6) channels and by passive diffusion. Intestinal Ca absorption is vitamin D-dependent via increased expression of TRPV6 in the proximal small intestine. Magnesium absorption is independent of vitamin D. The majority of active and passive Ca absorption occurs in the duodenum, together with passive Mg absorption. TRPV6 is highly expressed in the stomach and duodenum, TRPM6 in the terminal ileum and colon [26]. Recent modelling suggests the absorption of Ca and Mg is separated, in that Ca absorption occurs mainly in the duodenum and proximal small intestine, while Mg is absorbed mainly in the distal small intestine and colon [26].

Forty to ninety percent of intestinal Mg absorption is passive and varies in a linear manner with the dietary Mg content. When Mg intake is normal, active Mg transport only accounts for 30% of total Mg absorption [5]. With low Mg ingestion, active transport becomes more important [27]. Passive intestinal Mg absorption is dependent on electrochemical voltage [7, 25] and Mg concentration gradients across the enterocyte membrane [5]. Mutations in TRPM6 on chromosome 9q22 not only cause hypomagnesaemia, but can cause hypomagnesaemia with secondary hypocalcaemia (HSH), a syndrome with autosomal recessive inheritance. This is because Mg TRPM6, TRPM7 and Ca channels are similar with a fused alpha-kinase domain [28]. Chronic hypomagnesaemia in HSH can also cause hypoparathyroid hypocalcaemia [28]. TRPM7 channels may be required for movement of TRPM6 channel proteins to the plasma membrane. TRPM7 channel activity is regulated by intracellular concentrations of Mg and its nucleotides, and modulated via phosphotransferase kinase [25]. Control of TRPM6 and -7 expression or activity is influenced by angiotensin II, aldosterone, bradykinin, oestrogen, thiazide diuretics, epidermal growth factor (EGF) tacrolimus, and cholera toxin. PTH or 1,25 OH-Vitamin D have no effect on TRPM6 expression [25].

Different intestinal segments contribute unequally to Mg2+ absorption. In percentage of total input, the duodenum absorbs 11%, jejunum 22%, ileum 56% and large intestine 11% [7]. TRPM6 is expressed along the whole intestine and in the distal convoluted tubules, TRPM7 in most body tissues. The majority of TRPM6 expression in humans is found in the terminal ileum and colon [26]. Low serum Mg level usually induces increased expression of TRPM6 or -7 in enterocytes and TRPM6 in the renal tubules, leading to enhanced active transport in both the intestines and kidneys. The percentage of ingested Mg absorbed in the intestine can thus vary from 24–75%, depending on dietary Mg intake [13]. Rich dietary sources of Mg include nuts, whole grains, legumes, green leafy vegetables, milk and some mineral waters [29]. Magnesium absorption is enhanced by fermentable fibre, acidic luminal pH and by taking supplements with meals. [29–31] The average daily recommended elemental Mg dietary requirement for adult females and males is 320 mg and 420 mg, respectively. The amount of soluble Mg released from ligand binding sites in food is dependent on high levels of gastric acid. Bioavailability of organic forms of Mg (citrate/amino acid chelate) is greater than inorganic salts such as Mg oxide [29, 32].

Suggested risk factors or mechanisms for PPIH (serum level <0.70 mmol/L) include (i) impaired active absorption, caused by proton pump inhibitors (PPIs) interfering with TRPM6 and -7 channels [23], (ii) impaired passive absorption in the duodenum and proximal small bowe, as PPIs may increase the pH of the intestinal lumen which interferes with the expression of claudin/passive paracellular channels [7]; however decreased pH and increased Mg absorption in the distal ileum associated with PPI may be the reason why PPIH is not a more common phenomenon [33], (iii) rise in colonic pH due to local PPI inhibition of **H^+^/K^+^ ATPase**, leading to impaired colonic active and passive Mg transport [34], (iv) PPI-induced hypochlorhydria decreases ingested Mg salt solubility, (v) inadequate dietary intake, especially with a typical highly refined western diet (active transport is up-regulated as in the presence of low intake, passive absorption is inadequate) [25], and (vi) structural intestinal malabsorption syndromes, such as tropical sprue, coeliac disease or chronic pancreatitis. These are unlikely to cause PPIH as cessation of PPIs reverses hypomagnesaemia [17].

### Interaction between PPIs, magnesium and calcium absorption

The presence of defective TRPM6 in HSH syndrome led to the hypothesis of PPIs interfering with active intestinal Mg[2+] absorption. Many of the earlier studies suggested that PPIs inhibited active, rather than passive, Mg absorption [23, 24, 28]. This was also based on the observation that HSH patients, who had congenital defects in intestinal TRPM6 and active Mg transport, were able to maintain serum Mg levels by high dose oral Mg ingestion and passive absorption [28]. Perazella suggested that a fall in small intestinal pH may neutralize the glutamic and aspartic residues of TRPM6 and -7 on the intestinal brush border and renal tubular epithelial cells [23]. By changing the intestinal luminal pH, PPIs may induce defective function of the active transcellular transport channels TRPM6 and -7 [23]. It is not clear if PPI-induced hypomagnesaemia is an idiosyncratic reaction, or contributed to by mutations in TRPM6 and -7 [23]. This theory is supported by heterozygous mutations in TRPM7 causing malabsorption of Mg in mice [35]. In vitro trans-infected cell mutations in glutamate amino acid residues of TRPM6 and 7 transporters abolish their divalent cation selectivity and the ability of external protons to increase transcellular magnesium and calcium permeability. This may explain why a only small percentage of patients are susceptible to PPIs and luminal pH changes. There are, however, no reported sequence analysis for TRPM6 mutations in PPIH patients [5]. However, the existence of a susceptible, subclinical form of HSH has been questioned by Lainez, due to lack of a human phenotype in patients with heterozygous TRPM6 mutations [36]. Loss of active Mg[2+] absorption may be greater in patients receiving concurrent diuretics [23].

Luminal acidity along the gastrointestinal tract is important in Mg absorption as TRPM transporters and paracellular pores are pH sensitive [7]. There may be substantial inter- and intra-individual differences in gastrointestinal pH levels in healthy human subjects [37]. Under normal physiological conditions in murine and human studies, there is increasing pH and decreasing Mg solubility progressively along the duodenum, jejunum and ileum [38]. Small intestinal pH normally varies from a slightly acidic milieu in the duodenum (pH 5.6), to 6.2 in the second quartile (proximal jejunum), 6.68 in the third quartile, and 6.884 in the terminal ileum [39]. In the absence of gastric acid due to achlorhydria or PPIs, there is potentially less ionized Mg in the proximal intestinal and duodenal lumen available for active transport or passive diffusion [13]. After a week of 40 mg b.i.d. oral omeprazole in human subjects, Michalek *et al.* measured the intestinal pH using wireless motility capsule technology, showing that the pH actually fell from 6.9 to 6.4, 7.5 to 7.0 and 7.7 to 7.2 units, respectively, in the second, third and fourth quarters of the small intestine [33].

While some studies suggested increased pH and decreased Mg solubility in the duodenum and small bowel associated with PPI use [13, 38], Michalek *et al.* showed that PPI use caused with an average fall of 0.5 pH units in the mid-to-distal small intestine [33]. PPIs affect not only gastric proton pumps, but also extragastric sites such as the pancreatic duct and colonic H^+^/K^+^ ATPase pumps. Pancreatic duct proton pumps are necessary for active excretion of pancreatic bicarbonate, and colonic proton pumps for potassium absorption in the distal colon. It is suggested that these lower the pH in the duodenum and jejunum, and raise the pH in the distal colon. Decreased intestinal pH may also be related to intestinal bacterial overgrowth (SIBO), which could explain increased Mg absorption from the caecum in murine models and in humans. SIBO, as a side-effect of PPI use, has been well documented in the literature [40, 41]. This may be one of the reasons why hypomagnesaemia is not a universal phenomenon in long-term PPI use, due to variability between patients in intestinal pH, medication compliance or SIBO.

A Caco-2 (continuous cell line of human epithelial colorectal adenocarcinoma cells) in vitro colonic cell culture study by Thongon *et al.* suggested that omeprazole inhibited passive intestinal Mg2+, but not Ca2+ absorption [7]. The rate of passive intestinal Mg absorption is dependent on luminal acidity, transepithelial Mg concentration gradient and transepithelial electrical resistance (TEER) [7, 25]. The paracellular cation pores have a greater affinity for Mg at low pH. This is regulated by—and dependent on—the expression of negatively charged tight-junction membrane pore proteins claudin 7 and -12. PPIs may increase the luminal pH and TEER, which decreases paracellular Mg conductivity. PPIs also decrease the expression of claudin 7 and -12. PPI withdrawal restores apical acidity, claudin expression and passive Mg transport in the intestine [7]. This effect on passive Mg absorption was contrary to modelling by Bai *et al.* using *in vivo* human measurements of small intestinal pH [35].

PPIs may also affect colonic absorption of Mg. The pH in the caecum is usually acidic due to fermentation of carbohydrates, which is why fermentable fibre enhances colonic Mg absorption in humans and rodents [31]. PPI inhibition of H^+^/K^+^ ATPase colonic proton pumps may lead to an increase in distal colonic pH and indirectly decrease active colonic Mg absorption by TRPM6 and passive paracellular diffusion. This was suggested by Lameris *et al.* in a murine *in vivo* model [26, 34].

Magnesium is handled by the glomeruli filtering the 60–70% of plasma Mg that is not protein-bound. Serum Mg levels are largely determined by urinary Mg excretion. Re-absorption takes place throughout the nephron with 95% of filtered Mg being re-absorbed under normal physiological conditions. Renal excretion of the filtered Mg can be varied from 0.5–70%, depending on the plasma levels of Mg. The majority of Mg re-absorption (60–70%) is via passive paracellular tight junctions mediated by claudins in the thick ascending limb (TAL) of the loop of Henle. The distal convoluted tubule accounts for only 5–10% of Mg re-absorption via active transcellular transport involving TRPM6. This determines the final amount of excreted Mg in the urine [42, 43]. Renal TRPM6 can be up-regulated in the presence of hypomagnesaemia [25]. Hormones that are coupled to adenylate cyclase in the TAL, which affect passive Mg re-absorption, include PTH, calcitonin, glucagon, arginine vasopressin and beta-adrenergic agonists [25]. Patients with hypomagnesaemia demonstrate low 24-hr urinary Mg excretion due to avid re-absorption, with renal Mg excretion reduced to less than 1mEq/day [23]. After withdrawal of PPI, both serum and urinary Mg levels have been shown to return to normal [5, 44]. Apart from PPIH, all other forms of drug-induced hypomagnesaemia exhibit tubular Mg wasting [26].

Short-term PPI therapy does not cause severe hypomagnesaemia. In human subjects, short-term PPI therapy at most causes a 5% reduction in the serum level and a 1% reduction in intestinal absorption of Mg after 1 week of PPI treatment. This was based on the 0.5 unit drop in intestinal pH as measured by Michalek *et al.* [33, 35]. However, after 1 year of PPI treatment, this could potentially deplete Mg2+ stores by up to 80%, based on modelling by Bai *et al.* [35]. Body stores of Mg are primarily the bones (60%), in the hydroxyapatite crystal unit. Bone provides a reservoir to maintain serum Mg levels; however the Mg content of bone decreases with age and is not completely bioavailable during Mg deficiency [9, 14, 19, 20]. There is usually a long delay in the development of severe hypomagnesaemia with PPI use (approximately 5–10 years) as Mg stores are gradually depleted and not replenished [3, 10]. A highly refined western diet, deficient in Mg, may also contribute to PPIH, as low oral Mg ingestion renders active intestinal transport—rather than passive channels—critical for intestinal absorption [25, 35]. Prolonged PPI use, diuretic therapy, chronic incremental depletion of Mg stores and impaired Mg homeostasis with aging may explain the risk of PPIH being highest in elderly patients [3, 10, 25].

A large, retrospective, non-randomized study of 11 490 consecutive adult patients admitted to intensive care unit demonstrated that PPI use with diuretics was associated with a significant risk of hypomagnesaemia (OR 1.54). In patients not on diuretics, differences in serum Mg levels were not statistically significant [19]. A review, by Mackay *et al.*, of the 13 case studies in the literature from 2006–2010 indicated that patients with severe hypomagnesaemia were 68.8 ± 8.6 years old, on PPI therapy for a mean of 8.3 ± 3.5 years and 8 out of 13 were on diuretics at initial presentation [3]. Eight of these patients remained on PPI and this resulted in 18 emergency hospital admissions with complications of hypomagnesaemia [3].

There is debate about the frequency of PPIH with different PPIs. Luk *et al.* suggested this may be related to relative PPI potency, differences within US prescription practices and length of time each PPI has been commercially available [1]. Compared to omeprazole, the respective relative potencies of proton pump inhibition are 0.23, 0.90, 1.00, 1.60, and 1.82 for pantoprazole, lansoprazole, omeprazole, esomeprazole and rabeprazole, respectively [18]. When related to individual PPIs, the percentages of patients with PPIH in the FDA data were pantoprazole 1.7%, lansoprazole 0.8%, omeprazole 1.7%, esomeprazole 0.4% and rabeprazole 0.7%. Using esomeprazole as a reference, the corresponding PPIH relative risk (RR) was esomeprazole RR = 1, lansoprazole RR = 1.7, omeprazole RR = 3.8, pantoprazole RR = 4.3 and rabeprazole RR = 1.5. In our case series pantoprazole (lowest potency for acid suppression) caused hypomagnesaemia in 2 of 4 patients and esomeprazole in 2 of 4 patients. Thus there is a class effect in PPIH that is not completely explained by PPI potency alone [1].

Magnesium is a co-factor in over 300 enzyme pathways, including adenosine triphosphatase (ATPase). Hypomagnesaemia thus leads to deactivation of such important cellular functions as protein production, glucose and vitamin D metabolism and oxidative phosphorylation. Changes in intracellular Mg influence mitochondrial respiration. Magnesium acts as a neuromuscular stabilizer by interfering with acetyl choline release in the extracellular space and by inhibiting release of Ca by myocyte sarcoplasmic reticulum [45]. Hypomagnesaemia is associated with malnutrition, intestinal malabsorption, diarrhoea, alcoholism, cisplatin chemotherapy, EGFR inhibitors, diuretics, inherited renal tubular defects, interstitial nephritis, aminoglycosides and hyperaldosteronism [46].

Hypomagnesaemic hypocalcaemic hypoparathyroidism (HHHP) is related to impaired PTH release, defective PTH binding at tissue receptors (bone/renal) and increased peripheral metabolism of PTH. Magnesium is necessary for second messenger signalling via G proteins in the transduction of extracellular Ca levels by parathyroid cell Ca-sensing receptors. Impaired Mg-dependent adenyl cyclase generation of cyclic adenosine monophosphate (cAMP) leads to decreased release of PTH [47–49]. Magnesium is also necessary for the conversion of 25-hydroxyvitamin D to 1,25-dihydroxyvitamin D, which increases Ca absorption from the intestines and osteoclast activity in the bones. As such, a state of functional hypoparathyroidism occurs with hypomagnesaemia, resulting in hypocalcaemia and further neuromuscular irritability. Muscle twitching and weakness then progress to prolonged tetany, convulsions and cardiac arrhythmias [1].

In HHHP, the level of PTH is paradoxically low, as a low serum calcium level with normal Mg usually stimulates PTH secretion. The FDA data showed that 60% of subjects with PPI-induced hypomagnesaemia also had hypocalcaemia [1]. Hypomagnesaemia has been shown *in vitro* to affect bovine PTH secretion. Low Mg results in impaired PTH release, regardless of low Ca, which normally increases PTH release [50]. In humans, hypomagnesaemia reduces PTH secretion, as Mg is essential for parathyroid gland function by up-regulation of receptors such as CaSR, VDR and FGF23 [51]. Low levels of Mg stimulate PTH secretion, very low serum concentrations induce a paradoxical blockade of PTH release [20, 50]. PTH also increases renal Mg re-absorption; thus hypomagnesaemic hypoparathyroidism may progress with increasing tetany below a threshold Mg level [20, 52].

Gastric achlorhydria or PPIs may affect the bioavailability of Ca salts in ingested foods or supplements, leading to impaired Ca absorption and potential bone loss [17, 53–56]. This is particularly relevant in the absorption of calcium carbonate, which requires gastric acid for solubility. At neutral pH, only 1% of a 500 mg tablet of calcium carbonate is soluble. At pH 5.5, corresponding to duodenal pH, 86% is soluble. At pH 2.5, corresponding to normal gastric pH, solubility is 100% [56]. Other Ca salts, such as calcium gluconate or calcium citrate, are readily water-soluble and are not affected by gastric pH. There are differences in the amount of elemental Ca in organic calcium salts *vs.* inorganic calcium carbonate, which affects the tablet size, dose and ingestability. Calcium carbonate supplements have the highest amount of elemental calcium by weight (40%), but the lowest solubility. The amount of elemental Ca in calcium citrate is 21%, calcium lactate 14% and calcium gluconate 9% [56].

This effect of PPIs decreasing Ca solubility and absorption has been supported by animal studies [17, 54] but human studies have shown conflicting results [57]. Limitations of these studies include the method of measurement of Ca absorption, age/gender of subjects *vs.* controls, patients' comorbidities, post-menopausal achlorhydria, balance method applied, short term use (<12 days) of PPI, fasting state or lack of baseline Ca absorption measurement prior to PPI commencement [58]. One double-blind, placebo-controlled crossover study in 12 healthy young volunteers showed no short-term difference between placebo and omeprazole administered groups in terms of dual phase Ca isotope absorption or urinary Ca excretion [59]. Another randomized study in women showed a 41% decrease in calcium carbonate absorption with omeprazole. However, this was done in a fasting state, which is not entirely physiologically representative [30]. Gastric acid converts insoluble calcium carbonate to calcium chloride, which is highly soluble in water at neutral and even slightly alkaline pH [53]. Buffering of gastric acid in the duodenum occurs with pancreatic bicarbonate, such that the majority of active Ca transport in the duodenum occurs at a controlled, slightly acidic pH of 5–6 [59]. It is not established whether PPIs in humans affect the function of TRPV5 and -6, which are important in selective Ca absorption from the intestine and re-absorption from the renal tubules [43].

All three hypocalcaemic patients in our series improved their Ca levels after PPI withdrawal and Mg supplementation, without the need for concurrent Ca supplementation. Epstein showed that Ca levels recovered after discontinuation of PPI but before serum Mg had been corrected, while PTH levels rose only after recovery [20]. Ionized Ca absorption is also less affected than ionized Mg by the steric constraints of cation transmembrane transport. This is due to differences in Mg water affinity, hydrated Mg ion diameter and pore affinity for divalent cations [26, 27].

## CONCLUSION

There are no existing long-term prospective studies of PPIH, but there are increasing numbers of case reports, series and retrospective reviews [17]. This resulted in the FDA and TGA releasing safety announcements on PPIH in 2011. With long-term use of PPIs, it is important that clinicians are aware of the potential development of PPIH and related presentations. These range from no symptoms to lethargy and leg cramping to seizures and life-threatening arrhythmias [1–5]. Short-term PPI use is not usually associated with hypomagnesaemia, with onset of PPIH usually after five years of PPI use. Prompt removal of PPI and magnesium replacement can reverse PPIH and prevent re-hospitalizations and complications. Serum- and urinary 24-hour collections of Ca and Mg are both useful in monitoring PPIH. The syndrome of HHHP is commonly found in PPIH due to hypomagnesaemia interfering with PTH and Ca homeostasis, and should be considered in any patient presenting with cardiac arrhythmia, neuromuscular irritability or weakness [9, 19, 20, 35, 60].
